# Can gait patterns be explained by joint structure in people with and without radiographic knee osteoarthritis? Data from the IMI-APPROACH cohort

**DOI:** 10.1007/s00256-024-04666-8

**Published:** 2024-03-27

**Authors:** M. P. Jansen, D. Hodgins, S. C. Mastbergen, M. Kloppenburg, F. J. Blanco, I. K. Haugen, F. Berenbaum, F. Eckstein, F. W. Roemer, W. Wirth

**Affiliations:** 1 Department of Rheumatology & Clinical Immunology, University Medical Center Utrecht, HP G02.228 Heidelberglaan, 100 3584 CX Utrecht, The Netherlands; 2 Dynamic Metrics Limited, Codicote, UK; 3 Department of Rheumatology, Leiden University Medical Center, Leiden, The Netherlands; 4 Clinical Epidemiology, Leiden University Medical Center, Leiden, The Netherlands; 5 Departamento de Fisioterapia Y Medicina, Grupo de Investigación de Reumatología (GIR), INIBIC – Complejo Hospitalario Universitario de A Coruña, SERGAS. Centro de Investigación CICA, Universidad de A Coruña, A Coruña, Spain. Servicio de Reumatologia, INIBIC- Universidade de A Coruña, A Coruña, Spain; 6 Center for Treatment of Rheumatic and Musculoskeletal Diseases (REMEDY), Diakonhjemmet Hospital, Oslo, Norway; 7 Department of Rheumatology, AP-HP Saint-Antoine Hospital, Paris, France; 8 INSERM, Sorbonne University, Paris, France; 9 Department of Imaging and Functional Musculoskeletal Research, Institute of Anatomy and Cell Biology & Ludwig Boltzmann Institute for Arthritis and Rehabilitation (LBIAR), Paracelsus Medical University, Salzburg, Austria; 10 Chondrometrics GmbH, Freilassing, Germany; 11 Quantitative Imaging Center, Department of Radiology, Boston University School of Medicine, Boston, MA USA; 12 Department of Radiology, Universitätsklinikum Erlangen and Friedrich-Alexander-University Erlangen-Nürnberg (FAU), Erlangen, Germany

**Keywords:** Osteoarthritis, Structure, Pathology, Gait, ROM

## Abstract

**Objective:**

To determine the association between joint structure and gait in patients with knee osteoarthritis (OA).

**Methods:**

IMI-APPROACH recruited 297 clinical knee OA patients. Gait data was collected (GaitSmart®) and OA-related joint measures determined from knee radiographs (KIDA) and MRIs (qMRI/MOAKS). Patients were divided into those with/without radiographic OA (ROA). Principal component analyses (PCA) were performed on gait parameters; linear regression models were used to evaluate whether image-based structural and demographic parameters were associated with gait principal components.

**Results:**

Two hundred seventy-one patients (age median 68.0, BMI 27.0, 77% female) could be analyzed; 149 (55%) had ROA. PCA identified two components: upper leg (primarily walking speed, stride duration, hip range of motion [ROM], thigh ROM) and lower leg (calf ROM, knee ROM in swing and stance phases). Increased age, BMI, and radiographic subchondral bone density (sclerosis), decreased radiographic varus angle deviation, and female sex were statistically significantly associated with worse lower leg gait (i.e. reduced ROM) in patients without ROA (*R*^2^ = 0.24); in ROA patients, increased BMI, radiographic osteophytes, MRI meniscal extrusion and female sex showed significantly worse lower leg gait (*R*^2^ = 0.18). Higher BMI was significantly associated with reduced upper leg function for non-ROA patients (*R*^2^ = 0.05); ROA patients with male sex, higher BMI and less MRI synovitis showed significantly worse upper leg gait (*R*^2^ = 0.12).

**Conclusion:**

Structural OA pathology was significantly associated with gait in patients with clinical knee OA, though BMI may be more important. While associations were not strong, these results provide a significant association between OA symptoms (gait) and joint structure.

**Supplementary information:**

The online version contains supplementary material available at 10.1007/s00256-024-04666-8.

## Introduction

Gait alterations, such as a reduced range of motion (ROM) [1–7], have been observed in knee osteoarthritis (OA) patients, and are often considered to be associated with knee pain, as patients may alter their gait to minimize pain [8, 9]. However, it has not been explored to what extent such alterations could be related to the structural pathology of the joint, such as osteophyte formation, loss of cartilage, subchondral bone sclerosis, meniscal pathology, or synovitis [10]. For example, osteophyte formation has been shown to limit passive and active knee ROM, and as such might also affect gait [11–13]. While the relation between gait and radiographic joint structure has been analyzed using the Kellgren-Lawrence grade (KLG) in knee OA patients [14–16], it is not known how specific structural joint characteristics of knee OA, including those seen by MRI, are related to gait alterations. However, elucidation of this relationship is important, because understanding which structural alterations cause actual changes in symptoms and function as experienced by patients could provide important information on clinically relevant structural treatment targets. For this reason, the objectives of the current study were to determine the association between joint structure and gait in patients with clinical knee OA.

## Methods

### Participants

In the multicenter IMI-APPROACH (Applied Public–Private Research enabling OsteoArthritis Clinical Headway) cohort, 297 participants with clinical femorotibial (FT) knee OA, according to American College of Rheumatology (ACR) criteria, were included in five centers throughout Europe [17]. Machine learning algorithms were used to include participants with the greatest likelihood of progression in pain and/or structural joint pathology; the selection criteria and cohort profile have been published previously [17]. Exclusion criteria included predominantly patellofemoral OA, secondary knee OA (due to, e.g., severe leg deformity or inflammatory joint disease), generalized pain syndrome (fibromyalgia), and contraindications for undergoing MRI. At the first visit, the participants’ index knee was selected based ACR criteria [18]. If both knees met these, the more affected knee as indicated by the participant was selected as index knee or, if no difference was indicated, the right one was selected. Participants visited the hospital where data was collected, which included gait measurements, imaging and collection of clinical data such as the Western Ontario and McMaster Universities Arthritis Index (WOMAC) questionnaire.

The study was approved by the regional ethical committees and Institutional Review Boards (UMC Utrecht, Leiden University Medical Center, Complejo Hospitalario Universitario de A Coruña, AP-HP Saint-Antoine Hospital, and Diakonhjemmet Hospital) and was conducted in compliance with the study protocol, Good Clinical Practice (GCP), the Declaration of Helsinki, and the applicable ethical and legal regulatory requirements. All participants have received oral and written information and provided written informed consent. The study was registered under clinicaltrials.gov nr: NCT03883568.

### Gait measurements

**Fig. 1:**
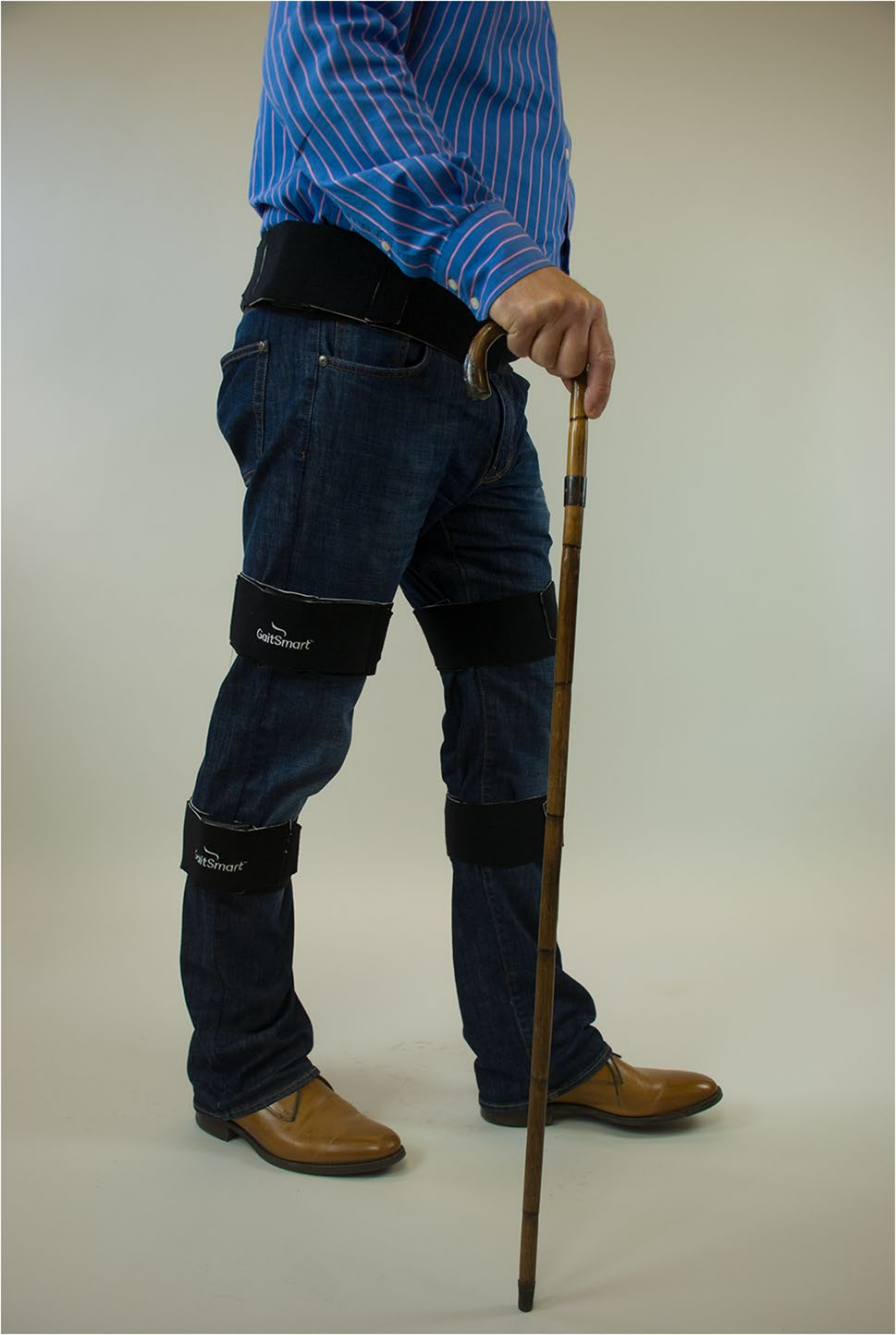
Example of GaitSmart system in use

Gait was assessed with the GaitSmart system, which uses six inertial measurement units (IMU), each comprising three tri-axial accelerometers and three tri-axial gyroscopes that allow for movement analysis in the sagittal and frontal plane [19]. The thigh sensors were attached along the sagittal plane of the thigh over the lateral aspect approximately 10 cm above the lateral joint line. The shin sensors were likewise attached over the widest part of the calf muscle taking different patient heights into consideration, see Fig. 1. The system has been validated in comparison with 3D analysis in an optical gait lab [19, 20]. The key finding was that the system was reproducible and there was no evidence of a difference in pelvic tilt and knee ROM, although the IMU system showed slightly less hip flexion.

After a 10 s stationary period for calibration, participants were asked to walk 15–20 m at their own speed and return, after which the IMUs were removed and connected to the laptop for analysis. The gait parameters assessed in the current study that were considered most relevant for the knee OA population, based on previous research [1, 3–7, 21–23], were walking speed (m/s) and stride duration (s), range of motion (ROM) of the hip and the knee in swing and stance phase. The ROM of the calf (tibia) and thigh (femur) segments in the sagittal plane was also quoted. All values were for the leg of the index knee (in ◦). The difference between the index leg and the contralateral leg was assessed as well, in order to use the less affected contralateral limb as reference for the target limb.

### Structural joint evaluation

The imaging protocol included 1.5 T or 3 T MRI scans and weight-bearing semi-flexed, posterior-anterior radiographs of the index knee [24]. Radiographs were performed according to the Buckland-Wright protocol and analyzed using KIDA software by one experienced observer, evaluating minimum joint space width (JSW, mm), femorotibial angle (FT angle, ◦ valgus), mean whole-joint subchondral bone density (SBD, mm Aluminum equivalent (mm Al eq) in reference to an aluminum step wedge), and total whole-joint osteophyte area (mm^2^) [25–28]. Kellgren-Lawrence grading (KLG) of both the index knee and contralateral knee was performed on the radiographs as well by an experienced rheumatologist.

The MRI protocol included sagittal and coronal intermediate weighted fat suppressed sequences, to perform semi-quantitative MRI Osteoarthritis Knee Scores (MOAKS) scoring by an experienced radiologist (FWR) of bone marrow lesions (BML, total number in FT joint), meniscal extrusion (score 0–3), meniscal tear (score 0–7), synovitis (score 0–3), and effusion (score 0–3) [29]. MOAKS scores are further explained in Supplementary Table S1. For the meniscal parameters, the maximum score across all regions was used [30]. Patellofemoral (PF) MOAKS scoring was performed as well (maximum PF cartilage loss and osteophyte scores used as sensitivity measures to correct for PF OA in the current study). The MRI protocol further included sagittal 3D SPGR sequences for quantitative analysis of the cartilage thickness in the femorotibial joint. The mean total FT joint cartilage thickness (FTJ ThC, mm) was determined from manual cartilage segmentations of the four femorotibial cartilages, which were performed by experienced readers with blinding to time point (Chondrometrics GmbH, Freilassing, Germany). All cartilage segmentations were quality controlled by an expert reader. Study-specific precision errors and two-year change in the IMI-APPROACH cohort have been published [24].

### Statistical analysis

While all IMI-APPROACH had clinical knee OA, only about half exhibited definite radiographic OA (ROA; KLG ≥ 2) in their index knee, as published previously [17, 31]. As preliminary analyses suggested differences in structure-gait associations between participants with and without ROA, participants with and without ROA were analyzed separately. Baseline demographics are presented for the two groups separately, and compared using Mann–Whitney *U* tests for continuous variables (as most were not normally distributed), and chi-square tests for categorical variables.

Gait parameters were compared between participants with and without ROA using independent *t*-tests, to help interpret gait parameters. Similarly, analyses of the differences between the index leg and contralateral leg were included only to better interpret gait parameters, but were not included in further analyses, since the focus of the current study was on the index leg for which structural evaluation was available.

Principle component analysis (PCA) was performed on the gait parameters, to reduce data and discover gait domains, before comparing these gait domains (principle components) with structural joint structure parameters. Spearman correlation coefficients were determined between gait domains and structural measures, to evaluate associations of individual structural measures with gait. For a more complete analysis of how joint structure is associated with gait, multivariable linear regression models were applied. Separate models were used, with each gait domain as dependent variable. All structure measures, which were selected based on expected clinical relevance, as well as age, sex, and BMI, were included as independent variables. From these full models, variables were removed one by one based on p-value with an exclusion threshold of *p* = 0.15, so that the final models would include only variables associated with gait. All models were adjusted for presence of contralateral ROA (fixed inclusion in the regression models). Sensitivity analyses were performed with additional inclusion of WOMAC pain and of PF OA parameters in the final models, to adjust associations for pain or presence of PF OA. A *p*-value of < 0.05 was considered statistically significant; no multiple testing correction was applied. Only patients with gait measurements, radiographs and MRI scans present were included.

## Results

### Participants

Of the 297 participants in IMI-APPROACH, 271 had gait measurements and imaging, and thus were included in the current analyses. Of those, 122 (45%) participants did not have ROA. Baseline characteristics and joint structure measures of both groups are shown in Table 1, identifying a greater BMI and age, stronger WOMAC pain, and generally more severe structural damage in the index and contralateral knee for participants with ROA.

**Table 1:** Baseline characteristics and joint structure parameters of participants with and without radiographic knee osteoarthritis

Parameter	Without ROA (*n* = 122)	With ROA (*n* = 149)	*P*-value
Age, years	66.5 (26.0–70.0)	68.0 (63.0–72.0)	**0.048**
Male sex, *n* (%)	33 (27)	30 (20)	0.180
BMI, kg/m^2^	26.5 (23.3–30.2)	27.1 (25.0–32.3)	**0.018**
WOMAC pain, 0–100*	75.0 (60.0–90.0)	70.0 (55.0–80.0)	**0.005**
Right index knee, *n* (%)	69 (57)	88 (59)	0.678
KLG, *n* (%)			** < 0.001**
-0	46 (38)	0 (0)	
-1	76 (62)	0 (0)	
-2	0 (0)	60 (40)	
-3	0 (0)	78 (53)	
-4	0 (0)	11 (7)	
Contralateral KLG, *n* (%)			** < 0.001**
-0	57 (49)	14 (10)	
-1	50 (43)	38 (26)	
-2	7 (6)	49 (34)	
-3	3 (3)	39 (27)	
-4	0 (0)	5 (3)	
Minimum JSW, mm	3.1 (2.6–3.7)	2.1 (0.8–3.1)	** < 0.001**
FT angle, ◦	-2.6 (-4.5 – -1.8)	-4.0 (-5.9 – -2.2)	**0.003**
SBD, mm Al eq	29.4 (27.0–33.2)	31.7 (27.5–35.1)	0.058
Osteophytes, mm^2^	8.2 (4.6–12.3)	26.3 (16.0–40.7)	** < 0.001**
FTJ, ThC, mm	3.3 (3.0–3.6)	3.0 (2.7–3.4)	** < 0.001**
Total # BML, *n* (%)			** < 0.001**
-0	87 (72)	44 (30)	
-1	21 (17)	32 (22)	
-2	12 (10)	14 (10)	
-3	0 (0)	18 (12)	
-4	1 (1)	25 (17)	
-5 +	0 (0)	15 (10)	
Meniscal extrusion, *n*			** < 0.001**
-0	67 (55)	16 (11)	
-1	40 (33)	36 (24)	
-2	13 (11)	52 (35)	
-3	1 (1)	44 (30)	
Meniscal tear, *n*			** < 0.001**
-0	70 (58)	25 (17)	
-1	0 (0)	0 (0)	
-2	19 (16)	7 (5)	
-3	10 (8)	2 (1)	
-4	11 (9)	13 (9)	
-5	0 (0)	0 (0)	
-6	9 (7)	87 (59)	
-7	2 (1)	14 (10)	
Synovitis, *n*			** < 0.001**
-0	53 (44)	35 (24)	
-1	56 (46)	78 (53)	
-2	11 (9)	32 (22)	
-3	1 (1)	2 (1)	
Effusion, *n*			** < 0.001**
-0	93 (77)	50 (34)	
-1	23 (19)	60 (41)	
-2	1 (1)	29 (20)	
-3	4 (3)	8 (5)	
PF cartilage loss, *n* (%)			** < 0.001**
-0	19 (16)	7 (5)	
-1	21 (17)	10 (7)	
-2	69 (57)	84 (57)	
-3	12 (10)	46 (31)	
PF osteophytes, *n* (%)			** < 0.001**
-0	73 (60)	17 (12)	
-1	45 (37)	75 (51)	
-2	3 (3)	37 (25)	
-3	0 (0)	19 (13)	

*BMI*, body mass index; *WOMAC*, Western Ontario and McMaster Universities Arthritis Index; *KLG *Kellgren-Lawrence grade; *JSW *joint space width; *FT *femorotibial; *SBD *subchondral bone density; *mm Al eq*; mm aluminum equivalent; *FTJ ThC *FT joint cartilage thickness; *BML *bone marrow lesion; *PF *patellofemoral. Median and interquartile range or *n* (%) are given. *Lower values indicate more severe pain

### Gait parameters

Gait parameters for participants with and without ROA are shown in Table 2. Participants with ROA had a significantly reduced ROM of the thigh, calf, knee in swing and stance phase (all *p* < 0.02), but not for the hip (Table 2). Participants with ROA showed a statistically significantly larger (negative) difference between index and contralateral leg than participants without ROA for all ROMs except the hip.

**Table 2:** Gait parameters for participants with and without radiographic knee osteoarthritis

GaitSmart Parameter	Without ROA (*n* = 122)	With ROA (*n* = 149)	*P*-value
Speed (m/s)	1.0 (0.2)	1.0 (0.2)	0.095
Duration (s)	1.1 (0.1)	1.1 (0.1)	0.868
*Index leg*			
ROM hip (◦)	34.1 (6.9)	32.7 (7.4)	0.108
ROM thigh (◦)	38.0 (6.0)	36.5 (5.5)	**0.013**
ROM knee (◦)	60.2 (7.6)	56.4 (6.6)	** < 0.001**
ROM calf (◦)	74.0 (5.8)	70.2 (6.6)	** < 0.001**
ROM knee stance phase (◦)	17.4 (4.6)	14.6 (4.7)	** < 0.001**
*Difference index leg and contralateral leg*			
ROM hip (◦)	-0.4 (4.3)	-0.8 (5.1)	0.495
ROM thigh (◦)	0.1 (3.1)	-0.8 (3.7)	**0.029**
ROM knee (◦)	-0.0 (4.3)	-1.7 (5.4)	**0.004**
ROM calf (◦)	0.6 (3.0)	-1.3 (3.4)	** < 0.001**
ROM knee stance phase (◦)	0.0 (4.1)	-1.7 (4.5)	**0.002**

*ROM *range of motion; *ROA *radiographic osteoarthritis

Two principal components/gait domains could be identified using PCA. The first PC consisted mostly of walking speed (weight coefficient 0.909), thigh ROM (0.801), hip ROM (0.787) and walking duration (-0.730), while knee ROM in swing phase (0.019), knee ROM in stance phase (0.403) and calf ROM (0.361) contributed less. The second PC consisted mostly of knee ROM in swing phase (0.926), knee ROM in stance phase (0.656) and calf ROM (0.854), while walking speed (0.334), thigh ROM (0.416), hip ROM (0.364) and walking duration (0.021) contributed less. As such, the first PC was considered to represent predominantly upper leg gait (upper leg gait domain), and the second PC was considered to represent lower leg gait (lower leg gait domain).

### Associations between gait and individual features of joint structure

No statistically significant Spearman correlations were observed between the upper leg gait domain and any of the individual features of joint structure, for either group. In participants without ROA, the KIDA FT angle (*ρ* = -0.21; 95%CI -0.38–-0.03; *p* = 0.021) was negatively correlated with the lower leg gait domain, while FTJ ThC (*ρ* = 0.21; 95%CI 0.03–0.38; *p* = 0.022) was positively correlated with lower leg gait, indicating that less severe varus malalignment and thinner cartilage were associated with worse gait (i.e. gait with reduced ROM values, more resembling that in ROA patients). In ROA participants, osteophyte size (*ρ* = -0.22; 95%CI -0.38–-0.06; *p* = 0.006) and meniscal extrusion (*ρ* = -0.23; 95%CI -0.38–-0.06; *p* = 0.006) were negatively correlated with lower leg gait, indicating that larger osteophytes and greater meniscal extrusion were associated with worse gait. All results including non-significant associations can be found in Supplementary Table S2.

### Regression models for relationship between gait, demographics and joint structure

In participants without ROA, higher BMI was significantly associated with worse upper leg gait (Table 3), although the *R*^2^ was low (0.05). For participants with ROA, male sex, higher BMI, and less synovitis were associated with worse upper leg gait, although again the *R*^2^ was low (0.12). Worse lower leg gait was associated with higher age and BMI, female sex, decreased varus deviation and higher SBD in participants without ROA (*R*^2^ = 0.24), and with female sex, higher BMI, larger osteophytes and increased meniscal extrusion in participants with ROA (*R*^2^ = 0.18). See Supplementary Figures S1 and S2 for full regression output, including all independent variables.

**Table 3:** Baseline characteristics and joint structure parameters included in final regression models

PC	Without ROA					With ROA				
	Variables	B (95%CI)	Beta	*P*	*R*^2^	Variables	B (95%CI)	Beta	*P*	*R*^2^
PC1 (upper leg)	BMI	-0.03 (-0.07 – 0.01)	-0.15	0.108	0.05	Sex	-0.30 (-0.70 – 0.10)	-0.12	0.135	0.12
						BMI	-0.05 (-0.08 – -0.02)	-0.28	< 0.001	
						Synovitis	0.19 (-0.03 – 0.42)	0.14	0.092	
PC2 (lower leg)	Age	-0.02 (-0.04 – 0.00)	-0.14	0.115	0.24	Sex	0.56 (0.19– 0.94)	0.23	0.004	0.18
	Sex	0.66 (0.33 – 1.00)	0.34	< 0.001		BMI	-0.04 (-0.06 – -0.01)	-0.20	0.010	
	BMI	-0.04 (-0.07 – -0.01)	-0.22	0.015		Osteophytes	-0.01 (-0.02 – -0.00)	-0.22	0.014	
	FT angle	-0.09 (-0.16 – -0.01)	-0.19	0.029		Meniscal Extrusion	-0.12 (-0.29 – 0.04)	-0.12	0.144	
	SBD	-0.04 (-0.07 – -0.00)	-0.20	0.034						

*PC *principal component; *ROA *radiographic osteoarthritis; *BMI *body mass index; *SBD *subchondral bone density

Including WOMAC pain in the models did not change results, except that BMI was no longer associated with upper leg gait in participants without ROA, and the *R*^2^ of the lower leg model improved markedly in participants without ROA (lower leg *R*^2^ = 0.32). Including PF cartilage loss and osteophytes in the models did not change results.

## Discussion

This study shows that structural joint pathology measured by radiography or MRI is statistically significantly associated with gait in people with clinical knee OA, both in those with and in those without ROA, although the associated parameters differed between groups. In all cases, structural joint pathology related to more severe OA was associated with more abnormal gait, as expected. This is with the exception of the FT angle, where more severe malalignment in varus direction resulted in a better gait pattern, though this was only the case in participants without ROA with somewhat less severe malalignment. While previous studies noted that varus movement during dynamic joint loading was associated with knee OA severity, but not with static leg alignment, it will be certainly worthwhile to investigate this association further in future studies [32, 33]. It is also important to note that for all models, R^2^ values were low, and clinical relevance of these findings should be studied further.

No structural joint pathology measures were associated with upper leg gait patterns in participants without ROA, while in participants with ROA, higher synovitis scores were surprisingly associated with better upper leg gait. Like the varus angle, this result is unexpected and cannot be directly explained, as the association remained significant and negative even after including pain in the model. However, after closer inspection, this result is strongly influenced by the only two patients with synovitis score 3. After exclusion of these two participants, synovitis is no longer significantly associated with upper leg gait (*p* = 0.25). As such, these results should be verified in a larger, well-balanced cohort.

Apart from joint structure, higher BMI and female sex were associated with worse gait in both the upper and lower leg, especially in participants with ROA. This is consistent with previous studies that also found these differences for both BMI and sex, to the largest extent in people with knee OA [34, 35]. The effect of BMI is possibly most important in OA patients, with every 1 kg greater load resulting in a 2.2 kg greater peak force at the knee joint [36]. This re-emphasizes the importance of weight control in knee OA patients.

The current post-hoc study was designed to evaluate whether gait patterns are associated with radiographic or MRI structural joint pathology. While association does not necessarily mean causation, these results indicate that, aside from BMI, subchondral bone density, osteophytes, and meniscal extrusion may be interesting structural targets. Prevention or treatment of these targets could have clinical impact by improving patients’ gait, but this should be confirmed in future studies, evaluating structure and gait changes. Further, it would be interesting to evaluate whether gait can predict structural changes in a future study, since gait may be relatively easy to modify and gait modification might (partially) allow preventing or slowing structural deterioration. Preliminary analyses using the current study setup showed no significant relation, but associations with separate gait measures instead of domains might yield different results and could be more interesting in this case, as individual gait modifications would be easier to interpret and implement than the gait domains as used in the current study.

This is the first study to find a significant association between OA symptoms, with gait as a measure for functional symptoms, and specific joint structure characteristics. Importantly, these results are statistically significant also when including WOMAC pain or PF OA measures in the model, indicating that the relationship exists even when accounting for pain or PF OA. A consistently significant relationship between patient-reported symptomatic outcomes, such as function and pain, and joint structural pathology that are characteristic of OA has been difficult to find, although some studies did find significant associations between pain and bone marrow lesions and, to a lesser degree, effusion [37–40]. For knee function, no clear relations have previously been found. The fact that SBD and osteophytes were the relevant joint structure measures included in the regression models in the current study further indicates the importance of bone in knee OA symptoms as experienced by patients, either directly (evaluated as patient-reported outcome measures) or indirectly (in this case, gait analysis).

This study had several limitations. First, while we correct for the presence of contralateral knee OA, the participants’ gait might be influenced by other factors as well. Having hip, ankle or foot OA, or other skeletal problems such as leg length discrepancy or hip dysplasia, could influence gait, and the analyses were not corrected for that. Second, 10 participants did not have ROA in their index leg, but did have ROA in their contralateral leg. This is possible because the index leg selection was based on clinical knee OA, not on structural characteristics. Since the regression models were corrected for the presence of contralateral ROA, these 10 participants were not excluded in the current study. Last, leg muscles could be expected to influence gait, but these were not evaluated in the IMI-APPROACH cohort and not included in the current analyses, which focused on OA-related joint structure only.

In conclusion, joint structure measures appear to be associated with lower leg gait characteristics in patients with clinical knee OA, although sex and especially BMI may be more important and *R*^2^ values were generally low. Overall, structural parameters indicating more severe knee OA were associated with more impaired gait, showing a significant association between functional gait and joint structure in OA, independent from pain.

## Supplementary information

Below is the link to the electronic supplementary material.

Supplementary file1 (DOCX 731 KB)

## Data Availability

All relevant data are available upon request by sending an email to the Rheumatology department of the UMC Utrecht (urrci@umcutrecht.nl). This is a non-author email address that allows for maintenance of long-term data accessibility.

## References

[1] Favre J, Jolles BM. Gait analysis of patients with knee osteoarthritis highlights a pathological mechanical pathway and provides a basis for therapeutic interventions. EFORT Open Rev. 2016;1:368. 28461915 10.1302/2058-5241.1.000051 PMC5367582

[2] Ro DH, Lee J, Lee J, Park JY, Han HS, Lee MC. Effects of knee osteoarthritis on hip and ankle gait mechanics. Adv Orthop. 2019;2019:9757369. 10.1155/2019/9757369 PMC6451827 31019809

[3] Nagano Y, Naito K, Saho Y, Torii S, Ogata T, Nakazawa K, et al. Association between in vivo knee kinematics during gait and the severity of knee osteoarthritis. Knee. 2012;19:628–32. 22192889 10.1016/j.knee.2011.11.002

[4] Zeni JA, Higginson JS. Differences in gait parameters between healthy subjects and persons with moderate and severe knee osteoarthritis: a result of altered walking speed? Clin Biomech (Bristol, Avon). 2009;24:372–8. 19285768 10.1016/j.clinbiomech.2009.02.001 PMC2715920

[5] Astephen JL, Deluzio KJ, Caldwell GE, Dunbar MJ. Biomechanical changes at the hip, knee, and ankle joints during gait are associated with knee osteoarthritis severity. J Orthop Res. 2008;26:332–41. 17960658 10.1002/jor.20496

[6] Burnett DR, Campbell-Kyureghyan NH, Topp R V, Quesada PM. Biomechanics of lower limbs during walking among candidates for total knee arthroplasty with and without low back pain. Biomed Res Int. 2015;2015:142562. 10.1155/2015/142562 PMC4480238 26171387

[7] Ismailidis P, Hegglin L, Egloff C, Pagenstert G, Kernen R, Eckardt A, et al. Side to side kinematic gait differences within patients and spatiotemporal and kinematic gait differences between patients with severe knee osteoarthritis and controls measured with inertial sensors. Gait Posture. 2021;84:24–30. 33260078 10.1016/j.gaitpost.2020.11.015

[8] Thorp LE, Sumner DR, Wimmer MA, Block JA. Relationship between pain and medial knee joint loading in mild radiographic knee osteoarthritis. Arthritis Care Res (Hoboken). 2007;57:1254–60. 10.1002/art.22991 17907211

[9] Henriksen M, Graven-Nielsen T, Aaboe J, Andriacchi TP, Bliddal H. Gait changes in patients with knee osteoarthritis are replicated by experimental knee pain. Arthritis Care Res (Hoboken). 2010;62:501–9. 20391505 10.1002/acr.20033

[10] Mitton G, Engelke K, Uk S, Laredo JD, Chappard C. A degenerative medial meniscus retains some protective effect against osteoarthritis-induced subchondral bone changes. Bone Rep. 2020;12:100271. 10.1016/j.bonr.2020.100271 PMC7251536 32478143

[11] Pottenger LA, Phillips FM, Draganich LF. The effect of marginal osteophytes on reduction of varus-valgus instability in osteoarthritic knees. Arthritis Rheum. 1990;33:853–8. 2363739 10.1002/art.1780330612

[12] Ozdemir F, Tukenmez O, Kokino S, Turan FN. How do marginal osteophytes, joint space narrowing and range of motion affect each other in patients with knee osteoarthritis. Rheumatol Int. 2006;26:516–22. 16025334 10.1007/s00296-005-0016-0

[13] Di Cesare PE, Haudenschild DR, Samuels J, Abramson SB. Pathogenesis of osteoarthritis. In: Firestein GS, Budd RC, Gabriel SE, McInnes IB, O'Dell JR, editors. Kelley and Firestein’s Textbook of Rheumatology. 10th ed. Amsterdam: Elsevier; 2017;1685–1704.

[14] Zeng X, Ma L, Lin Z, Huang W, Huang Z, Zhang Y, et al. Relationship between Kellgren-Lawrence score and 3D kinematic gait analysis of patients with medial knee osteoarthritis using a new gait system. Sci Rep. 2017;7:1–8. 28642490 10.1038/s41598-017-04390-5 PMC5481437

[15] Kwon SB, Ro DH, Song MK, Han HS, Lee MC, Kim HC. Identifying key gait features associated with the radiological grade of knee osteoarthritis. Osteoarthr Cartil. 2019;27:1755–60. 10.1016/j.joca.2019.07.014 31400498

[16] Elbaz A, Mor A, Segal G, Debi R, Shazar N, Herman A. Novel classification of knee osteoarthritis severity based on spatiotemporal gait analysis. Osteoarthr Cartil. 2014;22:457–63. 10.1016/j.joca.2013.12.015 24418677

[17] van Helvoort EM, van Spil WE, Jansen MP, Welsing PMJ, Kloppenburg M, Loef M, et al. Cohort profile: The Applied Public-Private Research enabling OsteoArthritis Clinical Headway (IMI-APPROACH) study: a 2-year, European, cohort study to describe, validate and predict phenotypes of osteoarthritis using clinical, imaging and biochemical mark. BMJ Open. 2020;10: e035101. 32723735 10.1136/bmjopen-2019-035101 PMC7389775

[18] Altman R, Asch E, Bloch D, Bole G, Borenstein D, Brandt K, et al. Development of criteria for the classification and reporting of osteoarthritis: Classification of osteoarthritis of the knee. Arthritis Rheum. 1986;29:1039–49. 3741515 10.1002/art.1780290816

[19] Zügner R, Tranberg R, Timperley J, Hodgins D, Mohaddes M, Kärrholm J. Validation of inertial measurement units with optical tracking system in patients operated with Total hip arthroplasty. BMC Musculoskelet Disord. 2019;20:52. 10.1186/s12891-019-2416-4 PMC6364439 30727979

[20] Monda M, Goldberg A, Smitham P, Thornton M, McCarthy I. Use of inertial measurement units to assess age-related changes in gait kinematics in an active population. J Aging Phys Act. 2015;23:18–23. 24306618 10.1123/japa.2012-0328

[21] van Helvoort EM, Hodgins D, Mastbergen SC, Marijnissen AK, Guehring H, Loef M, et al. Relationship between motion, using the GaitSmart^TM^ system, and radiographic knee osteoarthritis: an explorative analysis in the IMI-APPROACH cohort. Rheumatology. 2021;60:3588–3597. 10.1093/rheumatology/keaa809 PMC8328500 33367896

[22] McCarthy I, Hodgins D, Mor A, Elbaz A, Segal G. Analysis of knee flexion characteristics and how they alter with the onset of knee osteoarthritis: A case control study. BMC Musculoskelet Disord. 2013;14:1–7. 23692671 10.1186/1471-2474-14-169 PMC3663779

[23] Rahman J, Tang Q, Monda M, Miles J, McCarthy I. Gait assessment as a functional outcome measure in total knee arthroplasty: a cross-sectional study. BMC Musculoskelet Disord. 2015;16:66. 10.1186/s12891-015-0525-2 PMC4374376 25886558

[24] Wirth W, Maschek S, Marijnissen ACA, Lalande A, Blanco FJ, Berenbaum F, et al. Test–retest precision and longitudinal cartilage thickness loss in the IMI-APPROACH cohort. Osteoarthr Cartil. 2023;31:238–48. 10.1016/j.joca.2022.10.015 36336198

[25] Buckland-Wright JC, Ward RJ, Peterfy C, Mojcik CF, Leff RL. Reproducibility of the semiflexed (metatarsophalangeal) radiographic knee position and automated measurements of medial tibiofemoral joint space width in a multicenter clinical trial of knee osteoarthritis. J Rheumatol. 2004;31:1588–97. 15290740

[26] Buckland-Wright JC, Wolfe F, Ward RJ, Flowers N, Hayne C. Substantial superiority of semiflexed (MTP) views in knee osteoarthritis: a comparative radiographic study, without fluoroscopy, of standing extended, semiflexed (MTP), and schuss views. J Rheumatol. 1999;26:2664–74. 10606380

[27] Marijnissen ACA, Vincken KL, Vos PAJM, Saris DBF, Viergever MA, Bijlsma JWJ, et al. Knee Images Digital Analysis (KIDA): a novel method to quantify individual radiographic features of knee osteoarthritis in detail. Osteoarthr Cartil. 2008;16:234–43. 10.1016/j.joca.2007.06.009 17693099

[28] Jansen MP, Welsing PMJ, Vincken KL, Mastbergen SC. Performance of knee image digital analysis of radiographs of patients with end-stage knee osteoarthritis. Osteoarthr Cartil. 2021;29:1530–9. 10.1016/j.joca.2021.07.013 34343678

[29] Roemer FW, Jansen M, Marijnissen ACA, Guermazi A, Heiss R, Maschek S, et al. Structural tissue damage and 24-month progression of semi-quantitative MRI biomarkers of knee osteoarthritis in the IMI-APPROACH cohort. BMC Musculoskelet Disord. 2022;23:1–20. 36397054 10.1186/s12891-022-05926-1 PMC9670371

[30] Hunter DJ, Guermazi A, Lo GH, Grainger AJ, Conaghan PG, Boudreau RM, et al. Evolution of semi-quantitative whole joint assessment of knee OA: MOAKS (MRI Osteoarthritis Knee Score). Osteoarthr Cartil. 2011;19:990–1002. 10.1016/j.joca.2011.05.004 PMC4058435 21645627

[31] Jansen MP, Wirth W, Bacardit J, van Helvoort EM, Marijnissen ACA, Kloppenburg M, et al. Machine-learning predicted and actual 2-year structural progression in the IMI-APPROACH cohort. Quant Imaging Med Surg. 2023;13:3298–306. 37179936 10.21037/qims-22-949 PMC10167469

[32] Kutzner I, Trepczynski A, Heller MO, Bergmann G. Knee adduction moment and medial contact force – facts about their correlation during Gait. PLoS One. 2013;8:e81036. 10.1371/journal.pone.0081036 PMC3847086 24312522

[33] Specogna AV, Birmingham TB, Hunt MA, Jones IC, Jenkyn TR, Fowler PJ, et al. Radiographic measures of knee alignment in patients with varus gonarthrosis: effect of weightbearing status and associations with dynamic joint load. Am J Sports Med. 2007;35:65–70. 16998083 10.1177/0363546506293024

[34] Harding GT, Hubley-Kozey CL, Dunbar MJ, Stanish WD, Astephen Wilson JL. Body mass index affects knee joint mechanics during gait differently with and without moderate knee osteoarthritis. Osteoarthr Cartil. 2012;20:1234–42. 10.1016/j.joca.2012.08.004 22902710

[35] Phinyomark A, Osis ST, Hettinga BA, Kobsar D, Ferber R. Gender differences in gait kinematics for patients with knee osteoarthritis. BMC Musculoskelet Disord. 2016;17:1–12. 27072641 10.1186/s12891-016-1013-z PMC4830067

[36] Aaboe J, Bliddal H, Messier SP, Alkjær T, Henriksen M. Effects of an intensive weight loss program on knee joint loading in obese adults with knee osteoarthritis. Osteoarthr Cartil. 2011;19:822–8. 10.1016/j.joca.2011.03.006 21440076

[37] Yusuf E, Kortekaas MC, Watt I, Huizinga TWJ, Kloppenburg M. Do knee abnormalities visualised on MRI explain knee pain in knee osteoarthritis? a systematic Review. Ann Rheum Dis. 2011;70:60–7. 20829200 10.1136/ard.2010.131904

[38] Lo GH, McAlindon TE, Niu J, Zhang Y, Beals C, Dabrowski C, et al. Bone marrow lesions and joint effusion are strongly and independently associated with weight-bearing pain in knee osteoarthritis: data from the osteoarthritis initiative. Osteoarthr Cartil. 2009;17:1562–9. 10.1016/j.joca.2009.06.006 PMC2787856 19583959

[39] Hunter DJ, Guermazi A, Roemer F, Zhang Y, Neogi T. Structural correlates of pain in joints with osteoarthritis. Osteoarthr Cartil. 2013;21:1170–8. 10.1016/j.joca.2013.05.017 23973127

[40] Felson DT, Chaisson CE, Hill CL, Totterman SMS, Gale ME, Skinner KM, et al. The association of bone marrow lesions with pain in knee osteoarthritis. Ann Intern Med. 2001;134:541–9. 11281736 10.7326/0003-4819-134-7-200104030-00007

