# Supplementary material for: Can gait patterns be explained by joint structure in people with and without radiographic knee osteoarthritis? Data from the IMI-APPROACH cohort
Source: Skeletal Radiol. 2024 Mar 27;53(11):2409–16. doi: 10.1007/s00256-024-04666-8 (PMC11410921; doi:10.1007/s00256-024-04666-8)
Supplement: Supplementary file 1 — Supplementary file1 (DOCX 731 KB) [file 256_2024_4666_MOESM1_ESM.docx]

Supplementary Table S1: Meaning of MOAKS scores.

| **Score** | **Meaning** |
| --- | --- |
| *Meniscal extrusion* | |
| 0 | <2 mm |
| 1 | 2-2.9 mm |
| 2 | 3-4.9 mm |
| 3 | >5 mm |
| *Meniscal tear* | |
| 0 | Normal |
| 2 | Horizontal tear |
| 3 | Vertical tear |
| 4 | Complex tear |
| 6 | Partial maceration |
| 7 | Complete maceration |
| *Synovitis* | |
| 0 | None |
| 1 | Mild |
| 2 | Moderate |
| 3 | Severe |
| *Effusion* | |
| 0 | Physiologic amount |
| 1 | Small |
| 2 | Medium |
| 3 | Large |

Supplementary Table S2: All Spearman correlations between gait domains and individual structural parameters.

| **Without ROA** | | | | | | | | | | | |
| --- | --- | --- | --- | --- | --- | --- | --- | --- | --- | --- | --- |
| PC (gait domain) |  | Minimum JSW | FT angle | SBD | Osteophytes | FTJ ThC | Meniscal extrusion | Meniscal tear | Synovitis | Effusion | Total # BML |
| Upper leg | ρ | -.007 | .042 | -.101 | -.135 | -.067 | .009 | -.037 | .058 | .140 | .102 |
|  | P-value | .942 | .648 | .266 | .137 | .469 | .926 | .685 | .528 | .127 | .264 |
| Lower leg | ρ | .058 | **-.209*** | -.125 | -.086 | **.208*** | .119 | .069 | .134 | -.080 | -.047 |
|  | P-value | .523 | **.021** | .171 | .348 | **.022** | .194 | .453 | .142 | .386 | .612 |
| **With ROA** | | | | | | | | | | | |
| Upper leg | ρ | -.045 | -.069 | -.150 | -.069 | -.081 | .026 | -.009 | .123 | -.022 | .108 |
|  | P-value | .586 | .408 | .069 | .401 | .332 | .750 | .916 | .140 | .789 | .195 |
| Lower leg | ρ | -.022 | -.027 | -.048 | **-.224**** | .109 | **-.225**** | -.074 | .028 | -.113 | -.005 |
|  | P-value | .790 | .745 | .562 | **.006** | .193 | **.006** | .373 | .735 | .176 | .953 |

ROA: radiographic osteoarthritis; JSW: joint space width; FT: femorotibial; SBD: subchondral bone density; FTJ ThC: FT joint cartilage thickness; BML: bone marrow lesion.


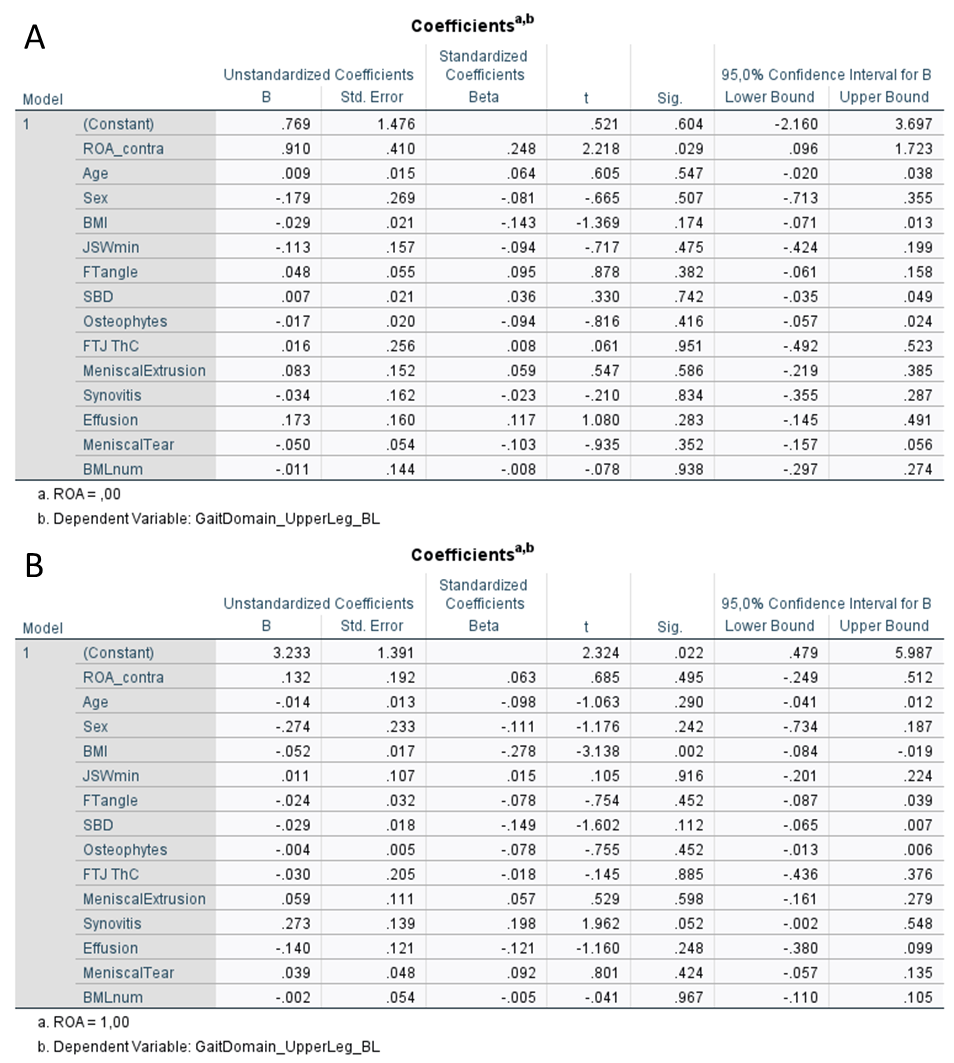


Supplementary Figure S1: Full regression output with the upper leg gait domain as dependent variable, for participants without radiographic osteoarthritis (ROA; A) and with ROA (B).


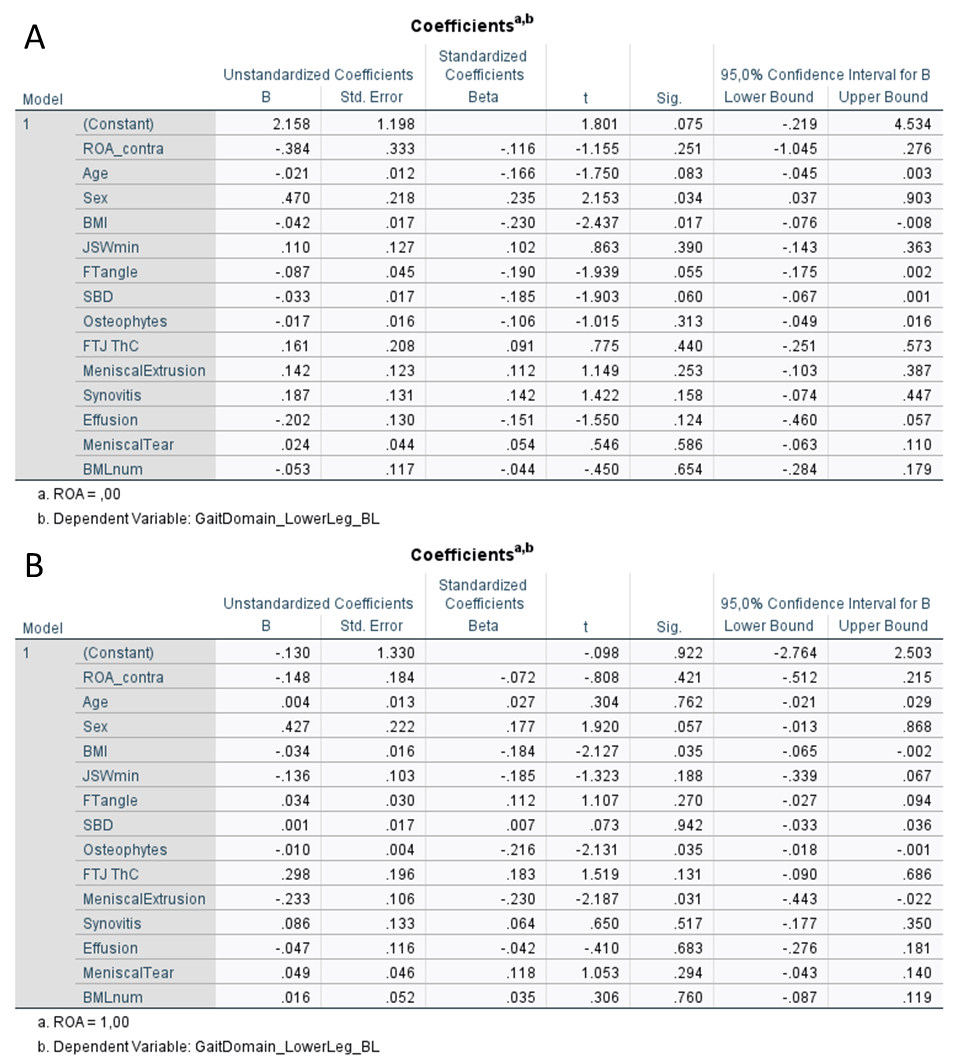


Supplementary Figure S2: Full regression output with the lower leg gait domain as dependent variable, for participants without radiographic osteoarthritis (ROA; A) and with ROA (B).
